# Distinct Local and Systemic Molecular Signatures in the Esophageal and Gastric Cancers: Possible Therapy Targets and Biomarkers for Gastric Cancer

**DOI:** 10.3390/ijms21124509

**Published:** 2020-06-25

**Authors:** Iwona Bednarz-Misa, Paulina Fortuna, Dorota Diakowska, Natalia Jamrozik, Małgorzata Krzystek-Korpacka

**Affiliations:** 1 Department of Medical Biochemistry, Wroclaw Medical University, 50-368 Wroclaw, Poland; iwona.bednarz-misa@umed.wroc.pl (I.B.-M.); paulina.fortuna@umed.wroc.pl (P.F.); natalia.jamrozik@student.umed.wroc.pl (N.J.); 2 Department of Gastrointestinal and General Surgery, Wroclaw Medical University, 50-368 Wroclaw, Poland; dorota.diakowska@umed.wroc.pl; 3 Department of Nervous System Diseases, Wroclaw Medical University, 51-618 Wroclaw, Poland

**Keywords:** cardia cancer, esophageal cancer, epithelial-mesenchymal transition, tight junction proteins, claudin-2, differential biomarkers, angiogenesis, metabolic reprogramming, inflammation

## Abstract

Gastric (GC) and esophageal (EC) cancers are highly lethal. Better understanding of molecular abnormalities is needed for new therapeutic targets and biomarkers to be found. Expression of 18 cancer-related genes in 31 paired normal-tumor samples was quantified by reversely-transcribed quantitative polymerase chain reaction (RTqPCR) and systemic concentration of 27 cytokines/chemokines/growth factors in 195 individuals was determined using Luminex xMAP technology. Only *Ki67*, *CLDN2,* and *BCLxL* were altered in GC while *Ki67*, *CDKN1A*, *ODC1*, *SLC2A1*, *HIF1A*, *VEGFA*, *NOS2*, *CCL2*, *PTGS2*, *IL10*, *IL10Ra,* and *ACTA2* were changed in EC. The relatively unaltered molecular GC landscape resulted from high expression of *BCLxL*, *CDKN1A*, *BCL2*, *Ki67*, *HIF1A*, *VEGFA*, *ACTA2*, *TJP1*, *CLDN2*, *IL7Ra*, *ODC1*, *PTGS2*, and *CCL2* in non-cancerous tissue. The *NOS2* expression and IL-4, IL-9, FGF2, and RANTES secretion were higher in cardiac than non-cardiac GC. Four-cytokine panels (interleukin (IL)-1β/IL-1ra/IL-6/RANTES or IL-1β/IL-6/IL-4/IL-13) differentiated GC from benign conditions with 87–89% accuracy. Our results showed increased proliferative, survival, inflammatory and angiogenic capacity in gastric tumor-surrounding tissue, what might contribute to GC aggressiveness and facilitate cancer recurrence. Further studies are needed to determine the *CLDN2* and *NOS2* suitability as candidate molecular targets in GC and cardiac GC, respectively, and discern the role of *CLDN2* or to verify IL-1β/IL-1ra/IL-6/RANTES or IL-1β/IL-6/IL-4/IL-13 usefulness as differential biomarkers.

## 1. Introduction

Gastric cancer (GC) and esophageal cancer (EC) are among the most lethal malignancies worldwide due to delayed diagnosis and lack of effective treatment modalities. While GC is the fifth most frequent diagnosed cancer and ranked third as a cause of cancer-related deaths [1,2,3], EC ranks seventh in incidence but sixth in cancer-related deaths [1]. The global effort to eradicate *Helicobacter pylori* infections, a main risk factor for GC, has caused a decrease in the incidence of a more common distal GC but concomitantly contributed to about seven-fold increase in incidence of its cardia subtype [4]. Cardia GC may share the etiology with non-cardia subtype or resemble esophageal adenocarcinoma [4]. *H. pylori* infections are responsible for chronic inflammation and oxidative stress in the gastric mucosa, leading to genetic instability and, consequently, to neoplastic transformation [5].

Radical surgery is the gold standard for treatment of solid tumors. Most often, however, GC and EC are recognized at an advanced stage, not amenable for curative resection and, thus, limiting therapeutic options to chemotherapy. Apart from serious side effects, the benefits of chemotherapy are rather disappointing [6]. Consequently, prognoses remain poor, with the five-year survival rates of ~20% in both GC [3] and EC [7]. Therefore, the urgent need for better understanding of the molecular mechanisms underlying the disease is emphasized in hope that it would lead to discovery of new therapeutic strategies, less toxic and more efficient, and ultimately improve survival [5]. As the mortality in GC and EC strongly depends on the disease stage at the time of diagnosis [2,7], non-costly and non-invasive tools allowing for early cancer detection are sought after as well.

Recent advances in biotechnology have facilitated a shift in research on biomarkers and molecular therapeutic targets from immunohistochemical determination of proteins, qualitative but semi-quantitative at best, towards unraveling the genetic and molecular anomalies underlying cancer, paving the way for personalized medicine [8]. Although delayed compared to other solid tumors, the “biomarker-driven cancer medicine“ approach in GC is gaining momentum [5]. Therefore, the aim of our study was comparative analysis of molecular signatures in GC at the local and systemic level with reference to cancer anatomical site (cardia and non-cardia GC) and as compared to EC. Locally, the expression of genes encoding key proteins relevant for cancer growth and progression was analyzed. Those included *Ki67* proliferation marker, *BCL2*, *BCLxL*, and *CDKN1A* (encoding p21^CIP1/WAF1^) pro-survival factors, *CCL2* (encoding monocyte chemoattractant protein (MCP)-1), *PTGS2* (encoding cyclooxygenase-2) and *NOS2* (encoding nitric oxide synthase-2) inflammatory factors, and *IL7* and *IL10* immune mediators and their receptors *IL7Ra* and *IL10Ra*. In addition, *HIF1A* (encoding hypoxia-inducible factor 1α) and *VEGFA* (encoding vascular endothelial growth factor (VEGF)-A) angiogenic factors, *ACTA2* (encoding smooth muscle α-2 actin; aSMA), *TJP1* (encoding zonula occludens (ZO)-1) and *CLDN2* (encoding claudin-2) epithelial-mesenchymal transition (EMT) markers, and *SLC2A1* (encoding glucose transporter GLUT1) and *ODC1* (encoding ornithine decarboxylase) metabolic reprogramming markers were quantified. At the systemic level, the concentration of 27 circulating cytokines, chemokines and growth factors was determined. Quantified cytokines included interleukin (IL)-1β, IL-1ra, IL-2, IL-4, IL-5, IL-6, IL-7, IL-8, IL-9, IL-10, IL-12(p70), IL-13, IL-15, IL-17A, interferon γ (IFNγ), IFNγ-induced protein 10 (IP-10), eotaxin 1 (EOX1), fibroblast growth factor 2 (FGF2), granulocyte colony-stimulating factor (G-CSF), granulocyte-macrophage colony-stimulating factor (GM-CSF), MCP-1, monocyte inflammatory protein (MIP)-1 α and β, platelet-derived growth factor (PDGF)-BB, RANTES, tumor necrosis factor α (TNFα), and VEGF-A.

## 2. Results

### 2.1. Local Expression of Cancer-Promoting Mediators

The local expression of genes encoding proteins facilitating cancer growth and progression was determined using real time (quantitative) polymerase chain reaction (PCR) methodology with SYBR green chemistry in 31 patient-matched samples of tumor and tumor-adjacent macroscopically normal tissue from GC (*n* = 15) and EC (*n* = 16) patients. Data on demography and pathology are presented in Table 1.

#### 2.1.1. Gastric Cancer

**Figure 1:**
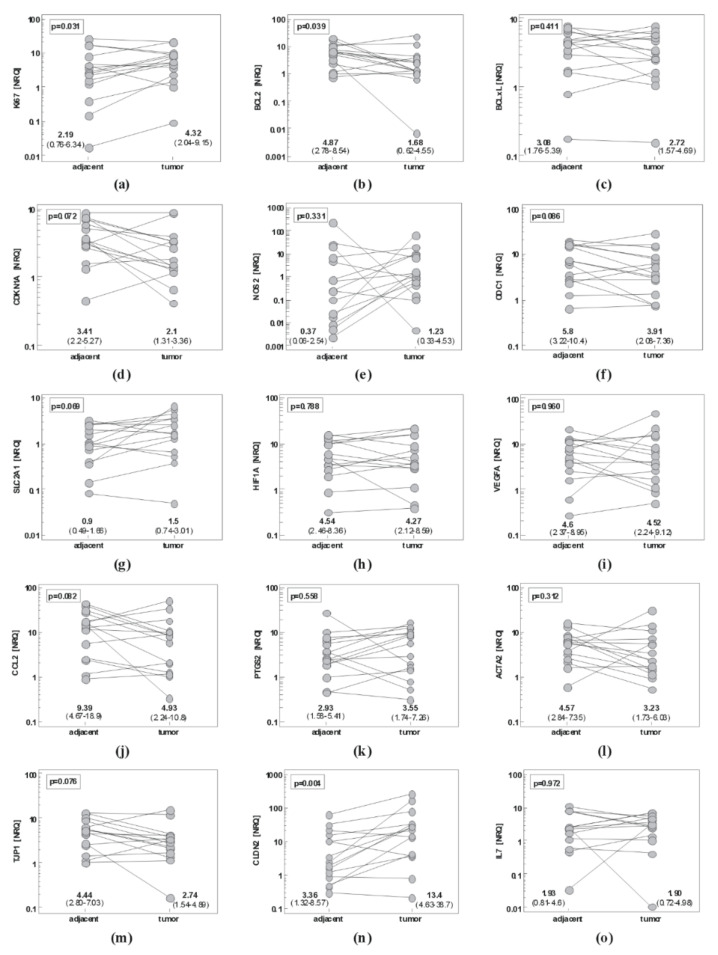

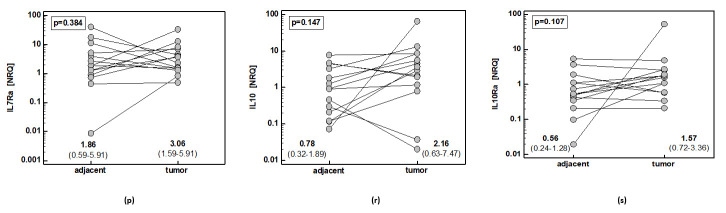
Pairwise analysis of local expression of cancer-promoting mediators in gastric mucosa: (**a**) *Ki67*; (**b**) *BCL2*; (**c**) *BCLxL*; (**d**) *CDKN1A*; (**e**) *NOS2*; (**f**) *ODC1*; (**g**) *SLC2A1*; (**h**) *HIF1A*; (**i**) *VEGFA*; (**j**) *CCL2*; (**k**) *PTGS2*; (**l**) *ACTA2*; (**m**) *TJP1*; (**n**) *CLDN2*; (**o**) *IL7*; (**p**) *IL7Ra*; (**r**) *IL10*; (**s**) *IL10Ra*. Data were analyzed as logs using *t*-test for paired samples and presented as geometric means of normalized relative quantities (NRQ) with 95% confidence interval (CI).

In GC, paired analysis showed significantly upregulated *Ki67* proliferation marker and tight junction protein *CLDN2* and downregulated anti-apoptotic *BCL2* in tumor as compared to non-cancerous tumor-adjacent tissue. Expression of *CDKN1A*, *ODC1*, *CCL2*, and *TJP1* tended to be lower and that of *SLC2A1* higher in tumors as well, but the differences did not reach statistical significance (Figure 1).

#### 2.1.2. Esophageal Cancer

**Figure 2:**
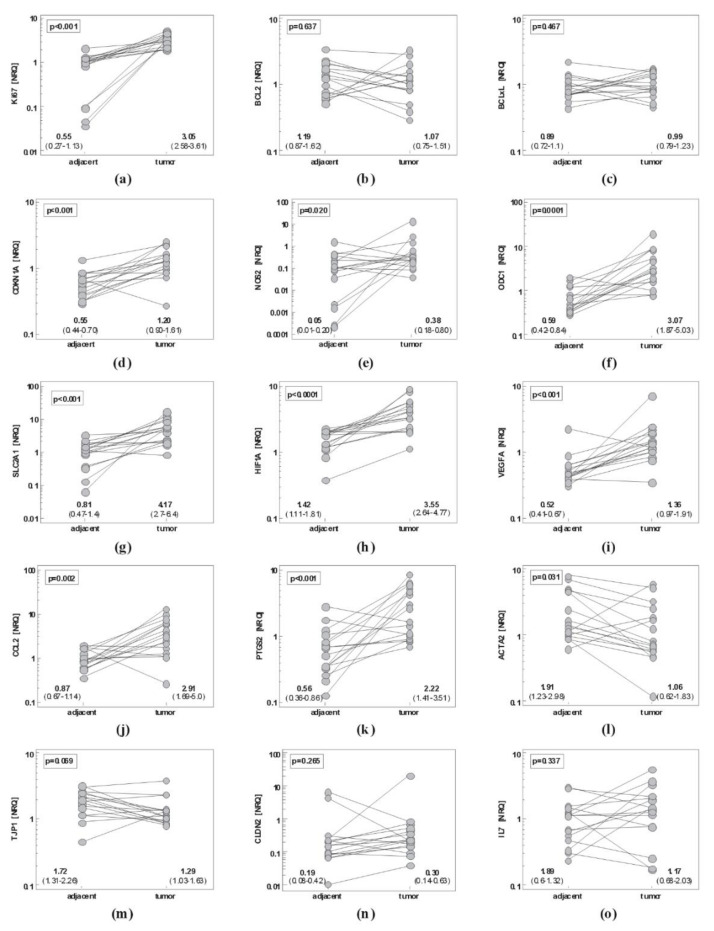

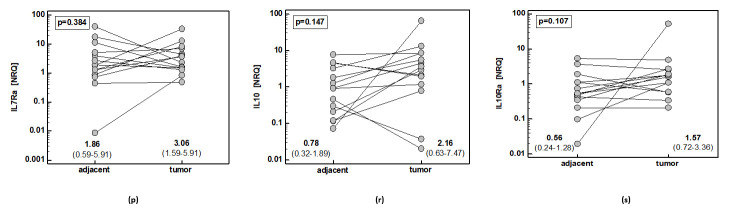
Pairwise analysis of local expression of cancer-promoting mediators in esophageal mucosa: (**a**) *Ki67*; (**b**) *BCL2*; (**c**) *BCLxL*; (**d**) *CDKN1A*; (**e**) *NOS2*; (**f**) *ODC1*; (**g**) *SLC2A1*; (**h**) *HIF1A*; (**i**) *VEGFA*; (**j**) *CCL2*; (**k**) *PTGS2*; (**l**) *ACTA2*; (**m**) *TJP1*; (**n**) *CLDN2*; (**o**) *IL7*; (**p**) *IL7Ra*; (**r**) *IL10*; (**s**) *IL10Ra*. Data were analyzed as logs using *t*-test for paired samples and presented as geometric means of normalized relative quantities (NRQ) with 95% confidence interval (CI).

In EC, paired analysis showed significantly upregulated *Ki67* and *CDKN1A*, markers of metabolic reprogramming *ODC1* and *SLC2A1*, mediators of angiogenesis *HIF1A* and *VEGFA*, mediators of inflammation and immunity *NOS2*, *CCL2*, *PTGS2*, *IL10* and its receptor *IL10Ra* and downregulated mesenchymal marker *ACTA2* in tumor as compared to non-cancerous tumor-adjacent tissue. In addition, epithelial marker *TJP1* tended to be decreased in esophageal tumors as well (Figure 2).

#### 2.1.3. Comparison of Gene Signatures in Gastric and Esophageal Cancers

##### Tumor-to-Adjacent Fold-Change in Expression

First, we investigated whether the differences in gene up- or downregulation (fold change in expression between tumor and adjacent tissue) differed between GC and EC (Table 1).

The expression of *Ki67*, *PTGS2*, and *SLC2A1* in tumors as compared to adjacent tissue was markedly more upregulated in EC than GC, by 2.8, 3.3, and 3.1-fold, respectively. There was no significant difference regarding anti-apoptotic *BCL2* and *BCLxL* but the difference in fold change in cell cycle regulator *CDKN1A*, downregulated in GC and upregulated in EC, was 3.5-fold. Fold change in *ODC1* and *CCL2*, downregulated in GC and upregulated in EC, was higher in EC than GC by 7.7 and 6.4-fold, respectively. The fold change in *HIF1A* and *VEGFA* was significantly different between GC and EC as well, by 2.7-fold in both cases (Table 2).

Of 15 GC patients, eight had cardia subtype and seven had non-cardia subtype of cancer. Comparison of gene expression signatures with respect to anatomical site showed *NOS2* to be more markedly upregulated in cardia GC (by 176-fold; mean fold change 36.7 in cardia vs. 0.21 in non-cardia, *p* = 0.022). Tumors from patients with cardia GC tended to have also higher *IL10* expression (by 7-fold; mean NRQ 5.7 vs. 0.8, *p* = 0.087).

##### Gene Expression in Tumors and Non-Cancerous Tissue

Subsequently, we compared gene expression (as normalized relative quantity) between gastric and esophageal tumors as well as between gastric and esophageal non-cancerous tumor-adjacent tissue (Table 3).

##### Independent Predictors of Gene Expression in Tumors and Non-Cancerous Gastric Tissue

Correlation analysis on genes differently expressed in GC and EC was conducted. First, significant associations were found in univariate analysis (Pearson correlation). Then, least squares multiple regression analysis was applied to identify variables independently from others associated with the expression of gene of interest. Partial (net) correlation coefficients (*r*_p_) were calculated for independent variables and model fit was expressed in terms of coefficient of determination (*R*^2^). Results are presented in Table 4 (non-cancerous tissue) and Table 5 (tumors).

The most striking difference between gastric and esophageal cancers was *CLDN2* expression, both regarding non-cancerous and tumor tissue. The expression of *Ki67* and *NOS2* was independently associated with *CLDN2* in non-cancerous adjacent tissue, explaining 83% in its variation, and *BCLxL* was independently associated with *CLDN2* in gastric tumors, explaining 64% in its variability. The expression of *PTGS2* was independently associated with *ACTA2* in non-cancerous tissue, explaining 38% in gene variability, and *BCL2* was independently associated with *ACTA2* in gastric tumors, explaining 39% in gene variability. The expression of *ACTA2*, *HIF1A*, and *VEGFA* was independently associated with *TJP1* in non-cancerous tissue, explaining 90% in its variability, and *BCL2*, *HIF1A*, *CDKN1A*, and *PTGS2* were independently associated with *TJP1* in gastric tumors, explaining 98% in gene variability.

The expression of *SLC2A1* and *HIF1A* was independently associated with *ODC1* in non-cancerous tissue, explaining 94% in its variability. In tumors, *HIF1A* was independently associated with *ODC1* expression, explaining 74% in its variability. The expression of *ODC1* was independently from other genes associated with *SLC2A1* in adjacent tissue, explaining 72% in its variability, and *Ki67* was independently associated with *SLC2A1* in tumors, explaining 63% in gene variability.

The expression of *TJP1* was independently associated with *VEGFA* in non-cancerous tissue, explaining 71% in gene variability, and *SLC2A1* was independently associated with *VEGFA* expression in tumors, explaining 59% in gene variability. The expression of *BCLxL*, *CDKN1A*, *CCL2*, and *ODC1* was independently from other genes associated with *HIF1A* in non-cancerous tissue, explaining 99% in its variability, and *CCL2* and *ODC1* remained independently associated with *HIF1A* in tumors, explaining 90% in its variability.

The expression of *CCL2* was independently associated with *PTGS2* in non-cancerous tissue, explaining 59% of variability in gene expression, and *CCL2*, *BCL2*, and *VEGFA* were independently associated with *PTGS2* expression in tumors, explaining 91% in gene variability. The expression of *PTGS2*, *BCL2*, and *IL7* (inversely) was independently associated with *CCL2* in non-cancerous tissue, explaining 88% in gene variability, and *HIF1A* and *BCL2* were independently associated with *CCL2* in gastric tumors, explaining 83% in its variation.

The expression of *BCLxL* and *CDKN1A* was independently associated with *Ki67* in non-cancerous tissue while *BCLxL* and *ODC1* in tumors, explaining 75 and 94% in gene variability, respectively. The expression of *HIF1A* was independently associated with *BCLxL* in non-cancerous tissue while with *CLDN2* and *Ki67* in tumors, explaining 81 and 91% in gene variability, respectively. The expression of *HIF1A* and *PTGS2* was independently associated with *BCL2* in non-cancerous tissue while with *TJP1* in tumors, explaining 77% and 78% in gene variability, respectively. The expression of *CCL2* was independently associated with *CDKN1A* in non-cancerous tissue and with *PTGS2* in tumors, explaining 87% and 92% in gene variability, respectively.

##### Impact of GC Pathological Stage on Fold Change in Gene Expression

The *Ki67* expression was upregulated in more advanced cancers—it correlated positively with TNM stage and presence of lymph node metastasis and tended to correlate with the extension of primary tumor. The *BCL2* and *BCLxL* expression tended to be less downregulated in gastric cancers with lymph node involvement and *BCL2* in more advanced primary tumors. The *CDKN1A* was less downregulated in more advanced and aggressive cancers as it is positively correlated with TNM stage, primary tumor extension, and histopathological grade, and tended to be upregulated in tumors with distant metastases present. The expression of *ODC1* was less downregulated in advanced cancers and a fold change in its expression correlated positively with TNM and lymph node metastasis and tended to correlate with the primary tumor extension as well. The expression of *HIF1A* was upregulated along with increasing TNM stage and in N1/2 cancers. Pro-inflammatory *CCL2* was less downregulated and *PTGS2* more upregulated in aggressive tumors. The fold change in *CCL2* expression correlated positively with the primary tumor extension and tended to be related to lymph node involvement. Fold change in expression of epithelial marker *TJP1* increased along with increasing TNM and the primary tumor extension. The gene was also less downregulated in GC patients with lymph node metastasis and tended to be upregulated in the presence of distant metastases. Immunosuppressive *IL10* was more pronouncedly upregulated in aggressive tumors (Table 6).

The correlation pattern observed for *TJP1* was counterintuitive and might be mediated by positive correlation between its expression and the expression of other genes (Table 4 and Table 5), also positively correlated with GC pathology (Table 6). Therefore, least squares multiple regression was applied to discern independent predictors of a fold change in *TJP1* expression. When co-examined with other genes, impact of TNM stage on *TJP1* lost significance as the association occurred to be mediated by *CDKN1A* and *HIF1A*. The *TJP1* association with tumor extension (T) lost significance as it was mediated by *BCL2* and *ODC1* and the *TJP1* association with lymph node metastasis (N) lost significance as it was mediated by *HIF1A*.

### 2.2. Systemic Cytokine Signatures in EC and GC

Systemic concentration of cytokines, chemokines and growth factors was determined using flow cytometry-based Luminex xMAP® technology in 195 individuals including 92 EC and 64 GC patients (32 with cardia and 32 with non-cardia subtypes), and 39 patients with benign conditions of esophagus and stomach. Data on demography and pathology are presented in Table 7.

Systemic concentration of IL-2, IL-15, and IL-17A was below the limit of detection in a great number of patients therefore those interleukins were excluded from further analysis.

#### 2.2.1. Systemic Concentration of Cytokines, Chemokines, and Growth Factors in Gastric and Esophageal Cancer and Benign Conditions

The GC patients had significantly higher concentration of IL-1β, IL-4, IFNγ, and PDGF-BB and lower of IL-1ra, IL-12(p70), IL-13, and MCP-1 than patients with EC or individuals with benign conditions of esophagus and stomach. In addition, they had higher IL-6 and G-CSF but lower RANTES than individuals with benign conditions and higher IL-9 and FGF2 but lower GM-CSF than EC patients (Table 8).

##### Cytokine Signatures Distinguishing between GC and Benign Conditions

In order to select cytokines distinguishing GC, data were log-transformed to allow for logistic regression analysis. Cytokines found significantly different between GC and benign conditions in univariate analysis (IL-1β, IL-1ra, IL-4, IL-6, IL-12(p70), IL-13, IFNγ, G-CSF, MCP-1, PDGF-BB, and RANTES) were entered as explanatory variables. Two methods were applied. In the stepwise approach, IL-1β, IL-1ra, IL-6, and RANTES were selected as independent GC predictors (cytokine panel 1). The model was characterized by a good fit (χ^2^ = 4.57, *p* = 0.803 in a Hosmer and Lemeshow test and Nagelkerke *R*^2^ = 0.51). In the backward approach, IL-1ra, IL-4, IL-13, and IL-6 were selected as independent GC predictors (cytokine panel 2). The model was characterized by a good fit (χ^2^ = 10.0, *p* = 0.264 in a Hosmer and Lemeshow test and Nagelkerke *R*^2^ = 0.54).

Receiver operating characteristics (ROC) curve analysis was conducted to evaluate individual cytokines and their panels as potential biomarkers in GC differentiating cancer patients from those with benign conditions. Individually, IL-1β, IL-1ra, and IFNγ had superior, but still only fair, overall accuracy. Only IL-1β had a Youden index higher than 0.5, indicative of superior combination of sensitivity and specificity. IL-1ra and G-CSF had superior sensitivity, allowing identifying patients with the disease, but accompanied by poor specificity. Cytokine panel 2 displayed equally good sensitivity, which, however, was accompanied by excellent specificity, minimalizing likelihood of false positives (Table 9).

##### Cytokine Signatures Distinguishing between GC and EC

In order to select cytokines distinguishing GC, cytokines found significantly different between GC and EC in univariate analysis (IL-1β, IL-1ra, IL-4, IL-9, IL-12(p70), IL-13, IFNγ, GM-CSF, MCP-1, PDGF-BB, and FGF2) were entered as explanatory variables into logistic regression analysis. IL-1ra, IL-4, IL-12(p70), and IL-13 were selected in a backward method and IL-1ra, IL-4, and IL-13 in a stepwise method. Both panels distinguished between GC and EC with similar accuracy. The area under ROC curve (AUC) was 0.79 (95%CI: 0.72–0.85) for the four-cytokine panel and 0.80 (0.73–0.86) for the three-cytokine panel.

##### 2.2.2. Cytokine Signatures Distinguishing between Cardia and Non-Cardia Subtypes of Gastric Cancer

**Figure 3:**
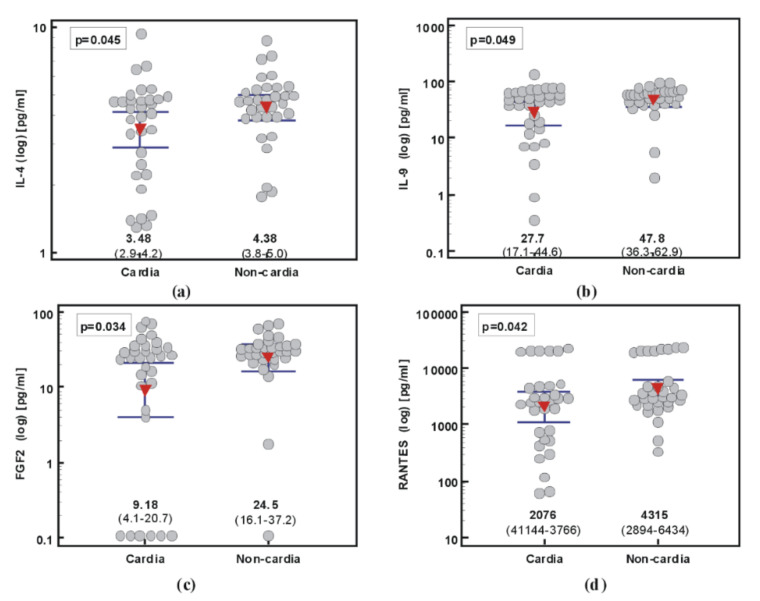
Systemic concentration of cytokines, chemokines, and growth factors in cardia and non-cardia subtypes of gastric cancer: (**a**) IL-4; (**b**) IL-9; (**c**) FGF2; (**d**) RANTES. Data presented as means (red triangles) with 95% confidence interval (whiskers) and analyzed using *t*-test for independent samples with Welch correction.

Comparison of cytokine concentration in patients with cardia and non-cardia gastric cancer showed similar systemic cytokine signatures in both cancer subtypes. Only IL-4 (by 1.3-fold), IL-9 (by 1.7-fold), FGF2 (by 2.7-fold), and RANTES (by 2.1-fold) were significantly higher, although borderline, in non-cardia GC (Figure 3).

In logistic regression, either FGF2 (backward method) or RANTES (stepwise method) were selected as independent predictors of non-cardia GC with comparable accuracy. The area under the ROC curve (AUC) was 0.63 (95%CI: 0.5–0.74) for FGF2 and 0.64 (0.51–0.75) for RANTES.

## 3. Discussion

Gastric and esophageal cancers are lagging behind others in implementing the idea of personalized medicine [5,6]. As frequently emphasized, there is an urgent need for discerning patterns of molecular abnormalities to facilitate discovery of novel targets and biomarkers [5,8]. Here, we examined expression patterns of 18 genes, encoding representative proteins relevant for cancer growth and progression. As compared to EC, in which expression of 12 genes was altered, gastric tumors had significantly upregulated expression of only two (*Ki67* and *CLDN2*) and downregulated one (*BCL2*). Qualitative differences were accompanied by quantitative, as *Ki67, PTGS2*, and *SLC2A* upregulation was significantly more pronounced in EC. Therefore, GC might appear to have relatively unaltered molecular landscape. However, the traditional analysis of fold change in expression ratio erroneously assumes “normality” of tumor-surrounding tissue. Actually, it has been argued that discerning molecular alterations happening in still non-transformed tissue is more informative on the processes leading to neoplastic transformation than the analysis based on already transformed cells and may pave the way to developing strategies for early cancer detection and/or primary chemoprevention [9]. Indeed, lack of gene upregulation and even their counterintuitive downregulation observed in GC seems to be associated with high gene expression in tumor-adjacent tissue. In fact, most genes were upregulated in “normal” gastric as compared to esophageal mucosa. The columnar epithelial cells lining the stomach are reportedly prone to inflammation and oxidative stress-induced damage [4], what would explain the particularly large difference in pro-inflammatory *CCL2* and *PTGS2*. The damage accumulation in cells holding high proliferative and survival capacity is particularly oncogenic [10]. Here we showed that, compared to esophageal apparently normal tissue, gastric mucosa had significantly upregulated expression of proliferation and survival markers *Ki67*, *BCL2*, *BCLxL*, and *CDKN1A*. This observation agrees well with increased risk of adenocarcinoma in chronic gastroesophageal reflux disease (GERD), associated with the replacement of squamous epithelium with columnar [11]. We further observed that non-cancerous gastric mucosa expressed markedly more pro-angiogenic factors (*HIF1A*, *VEGFA*, and *IL7* and its receptor *IL7Ra*) and EMT markers (*CLDN2*, *ACTA2*, and *TJP1*). Those observations add to the growing awareness that the macroscopically normal tumor-surrounding tissue might harbor molecular alterations [9,12,13,14,15,16]. Although not sufficient to change cell morphology, they are still of clinical relevance as the phenomenon of “molecular margin” is being argued to contribute to therapy failure and cancer recurrence following curative resection and/or to the occurrence of synchronous multiple tumors [12,13]. In addition to differences regarding non-transformed tissue, gastric tumors had higher than esophageal ones expression of *BCLxL*, *CDKN1A*, *VEGFA*, *ACTA2*, *CLDN2*, *TJP1*, and *IL7Ra*. Of note, markedly higher IL-7 protein upregulation in GC than EC has previously been reported [17].

Metabolic reprogramming of neoplastic cells with the accelerated glucose up-take is a recognized hallmark of cancer [18]. The overexpression of glucose transporter *SLC2A1*/GLUT-1 has been repeatedly shown in numerous solid tumors and associated with shorter overall and disease-free survival [19]. Here, *SLC2A1* was significantly upregulated only in EC, and was the sole gene overexpressed in esophageal as compared to gastric tumors. Cancer-type related variance in GLUT1 abundance is of clinical relevance as it directly correlates with the uptake of 18F-fluoro-2-deoxyglucose, a glucose analog used for cancer detection [20]. Therefore, cancers with low transporter expression are likely to pose a challenge for imaging employing positron emission tomography (PET). Corroborating our findings, Carvalho et al. [21] showed immunoreactivity for GLUT1 depends on the cancer anatomical site and histology, being low in gastric adenocarcinoma and present in cell cytoplasm rather than on the surface. Although EC has not been assessed, GLUT1 immunoreactivity in other squamous cell carcinomas was three to four times higher and evident on the cell surface. Others have postulated roles for GLUT1 beyond the transport of glucose, linking it with aggressive cancer behavior, high proliferation potential, and hypoxia [22,23]. Accordingly, *SLC2A1* expression correlated positively with markers of hypoxia and angiogenesis and mediators/indices of proliferation and survival. Moreover, factors indicative of high proliferative capacity—*Ki67* and *ODC1*—were independent predictors of *SLC2A1* expression in non-cancerous tissue and gastric tumor, respectively.

Ornithine decarboxylase, encoded by the *ODC1* gene, is a key enzyme in the polyamine biosynthesis pathway. Being exposed to harmful agents, the gastrointestinal tract mucosa had to be rapidly self-renewing. Polyamines play a crucial role in maintaining and controlling its proliferative, survival, migration, and angiogenic potential [24]. However, *ODC1* is a downstream target of the *MYC* oncogene and, thus, implicated in neoplastic transformation [25,26,27]. Here, we showed markedly higher *ODC1* expression in “normal” gastric than esophageal mucosa. This observation agrees well with the reported increase in polyamine concentration along the gastrointestinal tract [24]. Considering the enzyme role in mucosal healing, particularly high *ODC1* expression in gastric non-cancerous tissue is likely to be a response to overexpression of pro-inflammatory mediators. It has been shown that in the stomach, ornithine decarboxylase and polyamines are necessary for epithelial restitution and that the polyamine-mediated repair of the epithelial barrier involves upregulation of ZO-1 [24]. Accordingly, *ODC1* expression correlated positively with *TJP1* in both non-cancerous and tumor tissue. In line with a pro-proliferative character of polyamines, executed, among others, by polyamine-induced p21^CIP1/WAF1^ synthesis [24], *ODC1* expression correlated positively with *CDKN1A* and *Ki67.* Moreover, variability in *CDKN1A* expression independently predicted *ODC1* variation in non-cancerous tissue and variability in *Ki67*—in gastric tumors. Angiogenesis, manifested by upregulated *HIF1A* and *VEGFA* [24,28], is a part of the healing process of the gastric mucosa as well as a means of gastric cancer growth and dissemination. Studies with ornithine decarboxylase inhibitors have shown a stimulatory effect on angiogenesis, although, the underlying mechanisms remain obscure [24]. Still, cobalt-induced hypoxia in glioma cells resulted in increased expression of *ODC1* preceded by *HIF1A* upregulation [29]. Corroborating positive association between the enzyme and angiogenesis, *ODC1* correlated positively with *HIF1A* and *VEGFA*. More so, *HIF1A* was an independent predictor of *ODC1* expression in both non-cancerous and tumor tissue. Furthermore, despite apparent *ODC1* downregulation in gastric tumors, its expression positively correlated with cancer pathology—the TNM stage and, particularly, lymph node involvement.

A need for unraveling mechanisms underlying EMT in GC has been stressed and potential usefulness of EMT mediators as biomarkers and targets for preventative as well as curative interventions in GC has been suggested [30]. The EMT is associated with rearrangement in tight junction proteins, including downregulation of the epithelial marker *TJP1*/ZO-1 and upregulation of the mesenchymal marker *ACTA2*/aSMA [30]. While *TJP1* indeed tended to be downregulated in tumors as compared to adjacent tissue, *ACTA2* was downregulated as well, significantly so in EC. This, however, is probably caused by non-optimal tissue sampling from tumor bulk, as *ACTA2*-expressing myofibroblasts are located mostly at its border. Counterintuitively and contrary to literature data [31], *TJP1* expression correlated positively with GC pathology, but the association was apparent and mediated by other genes. Nonetheless, *VEGFA* and *HIF1* were independent predictors of *TJP1* expression. Correspondingly, others have shown the effect of VEGF-A on *TJP1*/ZO-1 to be inhibitory in endothelial but stimulatory in epithelial cells [32]. Here, *ACTA2* and *TJP1* were markedly more expressed in GC than EC, both in tumors and non-cancerous tissue. However, the most striking cancer-type related difference was associated with the expression of *CKDN2*, a gene encoding tight junction protein claudin-2. We found it upregulated by 44-fold in gastric tumors as compared to esophageal neoplasms and by 18-fold in non-cancerous tissue. Previously, upregulated claudin-2 immunoreactivity has been reported in esophageal squamous cell carcinoma [33] while data regarding GC are equivocal [34,35]. Claudin-2 is a pore forming protein but mounting evidence suggests that its role is not limited to regulating epithelial barrier permeability. The bulk of existing studies on claudin-2 in the gastrointestinal tract concerns colorectal cancer, where it is upregulated in response to IL-4 and IL-13 [16] and involved in promoting proliferation, survival, migration, colony formation, and drug resistance [16,36,37]. In addition, claudin-2 has been shown to facilitate self-renewal of colorectal stem-like cells. As these cells are held responsible for cancer recurrence following curative resection, claudin-2 has been proposed as a novel therapeutic target in colorectal cancer [38]. Scarce functional data in GC have shown that claudin-2 promotes migration but has no effect on the growth of gastric cancer cells [39]. Still, *CLDN2* expression in clinical samples examined here was independently associated with the expression of proliferation and survival markers, supporting its possible involvement also in improving cell viability. Taking into account that *CLDN2* expression has been shown to be downregulated by non-steroid anti-inflammatory drugs [39], it makes it a promising molecular target for cancer chemoprevention and warrants further in-depth functional studies on the protein.

In the present study, we compared the signatures of cardia and non-cardia subtypes of gastric cancer. Locally, cardia cancers were distinguished by comparatively high expression of *NOS2,* and tended to have seven-fold higher expression of *IL10*, which correlated positively with histological grade and, thus, with tumor aggressiveness. Taking into account inflammation and oxidative stress-promoting nature of NOS2 and immunosuppressive character of IL-10, their overexpression in cardiac cancers may contribute to generally worse prognosis associated with this subtype [40]. While this finding requires confirmation on a larger set of samples and on protein level, the notion was further supported by higher systemic concentrations of immunosuppressive IL-4, proangiogenic FGF2, and pro-inflammatory RANTES, observed here in patients with cardiac sublocation of the primary tumor. In addition, cardia GC was associated with elevated IL-9. The interleukin promotes inflammation [41] and plays a role in autoimmune diseases but its role in cancer is dichotomous [42].

One of the main reasons of high mortality accompanies EC and GC is their delayed detection, resulting from inconspicuous symptoms and lack of non-invasive diagnostic and differential biomarkers. Panels of cytokines have previously been shown to facilitate differential diagnosis in other cancers with superior accuracy [43]. Therefore, we aimed at determining the systemic cytokine signature of GC that would distinguish it from benign conditions. Individually, IL-1β had the highest diagnostic power as IFNγ and IL-1ra had a markedly worse Youden index, despite comparable accuracy. Both parameters were significantly improved for multi-cytokine panels. We built two sets—one included IL-1β (increased), IL-1ra (decreased), IL-6 (increased), and RANTES (decreased) and the other consisted of IL-1β, IL-6, IL-4 (increased), and IL-13 (decreased). Both panels were based on classic inflammatory cytokines IL-1 and IL-6, significantly more elevated in GC, despite the benign conditions analyzed here being inflammatory in nature as well. IL-1β is a prototypical pro-inflammatory cytokine induced by *H. pylori* infection, which further stimulate the expression of IL-6 and, concomitantly, of its non-functional analog IL-1ra, as a regulatory mechanism preventing hyperinflammation. The upregulation of IL-1β during *H. pylori* infection inhibits gastric acid secretion, facilitating further colonization of bacteria. It also increases secretion of gastrin, a hormone implicated in neoplastic transformation. Long-term, IL-1β oversecretion leads to the organ atrophy and adenocarcinoma [44,45]. Genetic studies have shown the risk for GC to depend on polymorphisms in the *IL1B* gene. Certain variants have been demonstrated to raise GC susceptibility by increasing IL-1β and reducing IL-1ra production [46]. Therefore, inclusion of IL-1β, IL-1ra, and IL-6 in the panels differentiating GC from benign conditions might be interpreted as a representation of more pronounced inflammation in GC. Interestingly, there was a difference in IL-4 and IL-13 between GC and benign conditions with elevated IL-4 and IL-13 in GC and benign conditions, respectively. Both interleukins are known to promote cancer development by interfering with anti-tumor immunity [47]. Recent findings, however, show that they may also support tumor cells directly, by facilitating their growth, survival, and migration [16,48,49]. They were both demonstrated to be elevated in gastric tumors as well as to upregulate the expression of *CLDN2* while downregulating that of *TJP1* in colonic cancer cells [16].

## 4. Materials and Methods

### 4.1. Patients

#### 4.1.1. Study Population—Local Molecular Signatures (Gene Expression)

Matched tissue samples (tumor and macroscopically normal tumor-adjacent) were collected intraoperatively from 51 cancer patients, admitted to the Department of Gastrointestinal and General Surgery of Wroclaw Medical University for curative resection of gastric adenocarcinoma (*n* = 15) or esophageal squamous cell carcinoma (*n* = 16). Patients with any severe systemic illness, with gross metastatic disease or subjected to radio- or chemotherapy were not included. Patients were subjected to a standard preoperative evaluation (blood work, physical examination, and imaging techniques, such as ultrasonography, computed tomography, and magnetic resonance). Cancers were rated pathologically using the 7th edition of the Union for International Cancer Control TNM system. In all cases, the resection margins have been confirmed to be tumor-free. Detailed population characteristics are depicted in Table 1.

#### 4.1.2. Study Population—Systemic Cytokine Signatures

The cohort of 195 patients was analyzed, including 39 controls (patients with benign conditions: gastritis, cardiospasmus, gastro-esophageal reflux disease, esophagitis) and 156 patients with histologically confirmed esophageal squamous cell carcinoma (*n* = 92) or gastric adenocarcinoma (*n* = 64). Among GC patients, 32 had adenocarcinomas of the gastric cardia and 32 had a non-cardia subtype (distal GC). All patients were admitted to the Department of Gastrointestinal and General Surgery of Wroclaw Medical University for the disease diagnosis and/or treatment (curative surgery or palliative treatment). Cancers were rated clinically using the 7th edition of the Union for International Cancer Control TNM system. Detailed population characteristics are depicted in Table 7.

#### 4.1.3. Ethical Considerations

The study protocol was approved by the Medical Ethics Committee of Wroclaw Medical University (signature number: KB 203/2016). The study was conducted in accordance with the Helsinki Declaration of 1975, as revised in 1983, and informed consent was obtained from all study participants.

### 4.2. Analytical Methods

#### 4.2.1. Sample Collection

##### Tissue Samples

Paired tissue samples were obtained intraoperatively and rinsed with saline prior their immersion in RNAlater solution (Ambion Inc., Austin TX, USA). Tissue samples were then stored at −80 °C until RNA isolation.

##### Serum Samples

Peripheral blood was collected by venipuncture into BD Vacutainer CAT tubes (Becton Dickinson, Plymouth, UK) and clotted for 30 min at room temperature (RT). Samples were subsequently centrifuged at 1500 × *g* for 10 min at RT. Collected sera were aliquoted and stored at −45 °C until examination. Blood samples were taken upon admission, prior to any treatment.

#### 4.2.2. Transcriptional Analysis

##### Tissue Homogenization

Tissue samples (up-to 40 mg) were homogenized in lysis buffer (provided as a part of PureLink™ RNA Mini Kit) with β-mercaptoethanol (Sigma-Aldrich, St. Louis, MO, USA) using Fastprep 24 Homogenizer (MP Biomedical, Solon, OH, USA) and ceramic spheres.

##### RNA Isolation

Total RNA was isolated using phenol-chloroform extraction followed by purification with PureLink™ RNA Mini Kit (Thermo-Fisher Scientific, Waltham, MA, USA) involving on-column digestion of genomic DNA with PureLink™ DNase Set (Thermo-Fisher Scientific). Purified RNA isolates were quantified using NanoDrop 2000 (Thermo-Fisher Scientific). RNA purity was determined by calculating ratios of absorbance at 260, 280, and 230 nm. RNA integrity was evaluated using the Experion platform, incorporating LabChip microfluidic technology, and Experion RNA StdSens analysis kits (BioRad, Hercules, CA, USA). RNA quality indicator (RQI) score was calculated for each RNa sample and only RNA isolates with RQI ≥ 7, indicative of good RNA quality, were used for reversely-transcribed quantitative polymerize chain reaction (RT-qPCR).

##### cDNA Synthesis

Aliquots containing 1000 ng of RNA were reversely transcribed using C1000 termocycler (BioRad) and iScript™ cDNA Synthesis Kit (BioRad), following the manufacturer’s instructions.

##### Quantitative (Real-Time) PCR

Quantitative PCRs were conducted using CFX96 Real-Time PCR system (BioRad) and SsoFast EvaGreen® Supermix (BioRad). The cycling conditions were as follows: 30 sec activation at 95 °C, 5 sec denaturation at 95 °C, annealing/extension for 5 sec at 61 °C, 40 cycles, followed by melting step (60–95°C with fluorescent reading every 0.5 °C). Reaction mixture contained 2 µL of cDNA (diluted 1:5), 10 µL of 2× SsoFast EvaGreen® Supermix, 1 µL of each 10 nM forward and reverse target-specific primers, and water up to 20 µL. Primers were synthesized by Genomed (Warsaw, Poland) and their sequences are presented in Table 10. Primers’ specificity was tested by melting curve analysis and an electrophoresis in a high-resolution agarose (SeaKem LE agarose from Lonza, Basel, Switzerland) in TBE with SYBR Green (Lonza) detection.

##### Expression Calculation and Normalization Strategy

Technical replicates were averaged prior analysis. Geometric mean of all Cq values across all samples was calculated and subtracted from individual sample Cq (ΔCq). Subsequently, ΔCq values were linearized by 2^^ΔCq^ conversion and normalized to *GAPDH,* serving as an internal control. The obtained values are referred to as a normalized relative quantity (NRQ) [50] and subjected to statistical analysis.

#### 4.2.3. Serum Cytokine Quantification

Serum concentration of 27 cytokines, chemokines, and growth factors was quantified using the BioPlex 200 platform (Bio-Rad), incorporating Luminex xMAP® technology, allowing for simultaneous quantification of multiple analytes in real-time, and Bio-Plex Pro™ Human Cytokine, Chemokine, and Growth Factor Magnetic Bead–Based Assays. This flow cytometry-based method utilizes magnetic microspheres conjugated with monoclonal antibodies and fluorescent reading. All analyses were conducted in duplicates following manufacturer’s instructions. Standard curves were drawn using 5-PL logistic regression and the data were analyzed using BioPlex Manager 6.0 software.

### 4.3. Statistical Analysis

Normality of distribution was evaluated using the Kolmogorov–Smirnov test. Homogeneity of variances was tested using the Levene test. Paired data were analyzed using the *t*-test for paired samples. Two-group comparisons were conducted using *t*-test for independent samples, with Welch correction in case of unequal variances, and resulting data are presented as geometric means with 95% confidence interval (CI). Multi-group comparisons were conducted using either one-way ANOVA on log-transformed data, with Tukey–Kramer post-hoc test, or Kruskal–Wallis *H* test, with the Conover post-hoc test. Resulting data are presented as, respectively, means or geometric means with standard deviation (SD) or 95%CI or medians with interquartile range. Frequency analysis was conducted using Fisher’s exact test (2 × 2) or χ^2^ test. Correlation analysis was conducted using Spearman’s rank correlation test (*ρ*) or Pearson correlation test.

Least squares multiple regression (stepwise method) was used to discern independent predictors of gene expression. Variables were entered into the model if *p* < 0.05 and removed if *p* > 0.1. Partial correlation coefficients (*r*_p_) with the effect of co-variables removed were calculated. Goodness-of-fit of the built regression model is expressed in terms of coefficient of determination (*R*^2^).

Logistic regression, stepwise and backward method, was applied to select independent explanatory variables. Variables entered the model if *p* < 0.05 and was removed if *p* > 0.1. Goodness-of-fit of a build model was determined by the Nagelkerke coefficient of determination (*R*^2^) and the Hosmer and Lemeshow test (tests for lack of fit; therefore, *p* > 0.05 is indicative of a model fit). Calculated probabilities from logistic regression were subsequently used as dependent variable in ROC curve analysis. The ROC curve analysis was applied to determine the diagnostic power (or to test the strength of association) of individual and multiple cytokines. Their ability to distinguish GC was assessed in terms of overall accuracy expressed as AUC (in %) and sensitivity and specificity at a given cut-off, summarized as Youden index (*J* = sensitivity + specificity − 100).

All calculated probabilities were two-tailed. The *p* values ≤0.05 were considered statistically significant. The entire analysis was conducted using MedCalc Statistical Software version 19.2 (MedCalc Software Ltd, Ostend, Belgium; https://www.medcalc.org; 2020).

## 5. Conclusions

Taken together, our results show increased proliferative, survival, inflammatory, and angiogenic capacity in gastric tumor-surrounding tissue as compared to esophageal non-cancerous mucosa. It might contribute to GC aggressiveness and facilitate cancer recurrence following curative tumor resection. Distinct molecular patterns between cardiac and non-cardiac GC with upregulated expression of pro-inflammatory and pro-oxidative *NOS2* and immunosuppressive *IL10* might contribute, in turn, to worse prognosis associated with cardiac GC subtype. We also showed distinct systemic cytokine signatures in gastric and esophageal cancers and their benign conditions, with cytokine panels consisting of IL-1β/IL-1ra/IL-6/RANTES or IL-1β/IL-6/IL-4/IL-13 holding promise as differential biomarkers in GC. The striking upregulation of *CLDN2* in GC and *NOS2* in the cardiac GC subtype warrants further studies on a larger cohort and with concomitant protein assessment to determine their suitability as candidate molecular targets. Functional studies discerning the role of *CLDN2* in GC are needed as well.

## Figures and Tables

**Table 1:** Characteristics of study population for analysis of local molecular signatures.

Characteristics:	EC	GC	*p*
*n*	16	15	−
Sex (F/M), n	6/10	4/11	0.704 ^1^
Age (yrs.), mean ± SD	63.3 ± 7	66.0 ± 12	0.465 ^2^
Stage (I/II/III/IV)	0/5/9/2	2/3/7/3	0.404 ^3^
Primary tumor, T (1/2/3/4)	0/5/8/3	1/1/9/4	0.279 ^3^
Lymph node metastasis, N (no/yes)	8/8	5/10	0.472 ^1^
Distant metastasis, M (no/yes)	14/2	12/3	0.654 ^1^
Histological grade, G (1/2/3/x)	5/7/4/0	1/6/7/1	0.209 ^3^

*n*, number of observations; F/M, female-to-male ratio; yrs., years; SD, standard deviation; ^1^, Fisher’s exact test; ^2^, *t*-test for independent samples with Welch correction for unequal variances; ^3^, Chi-squared test; EC, esophageal squamous cell carcinoma; GC, gastric adenocarcinoma.

**Table 2:** Comparison of fold change in gene expression between tumor and non-cancerous tumor-adjacent tissue in gastric and esophageal cancer.

Gene	Fold-Change (Tumor-to-Adjacent)		*p*
	GC	EC	
*Ki67*	1.97	5.52	0.032 ^1^
*BCL2*	0.35	0.90	0.078 ^2^
*BCLxL*	0.88	1.10	0.267 ^1^
*CDKN1A*	0.62	2.18	<0.001 ^1^
*NOS2*	3.29	8.12	0.527 ^1^
*ODC1*	0.68	5.17	<0.0001 ^1^
*SLC2A1*	1.66	5.15	0.017 ^1^
*HIF1A*	0.94	2.51	0.001 ^1^
*VEGFA*	0.98	2.62	0.025 ^2^
*CCL2*	0.52	3.34	<0.001^1^
*PTGS2*	1.21	4.01	0.014 ^1^
*ACTA2*	0.71	0.56	0.564 ^1^
*TJP1*	0.62	0.75	0.496 ^1^
*CLDN2*	3.98	1.61	0.128 ^1^
*IL7*	0.98	1.32	0.642 ^2^
*IL7Ra*	1.65	1.95	0.805 ^1^
*IL10*	2.76	3.9	0.616 ^2^
*IL10Ra*	2.8	1.74	0.467 ^2^

Data presented as expression ratio (fold change) between tumor and non-cancerous tumor-adjacent tissue and analyzed on log-transformed data using ^1^ *t*-test for independent samples or ^2^ *t*-test for independent samples with Welch correction.

**Table 3:** Expression patterns of cancer-promoting genes in gastric cancer as compared to esophageal and colorectal cancer.

Gene	Non-Cancerous Tissue (NRQ)			Tumor (NRQ)		
	GC	EC	*p*	GC	EC	*p*
*Ki67*	2.19	0.55	0.027 ^1^	4.32	3.05	0.347 ^2^
*BCL2*	4.87	1.19	0.0001 ^2^	1.68	1.07	0.372 ^2^
*BCLxL*	3.08	0.89	<0.001 ^2^	2.72	0.99	0.002 ^2^
*CDKN1A*	3.41	0.55	<0.0001 ^2^	2.1	1.2	0.038 ^1^
*NOS2*	0.37	0.05	0.073 ^1^	1.23	0.38	0.100 ^1^
*ODC1*	5.8	0.59	<0.0001 ^1^	3.91	3.07	0.518 ^1^
*SLC2A1*	0.9	0.81	0.778 ^1^	1.5	4.17	0.011 ^1^
*HIF1A*	4.54	1.42	0.001 ^2^	4.27	3.55	0.609 ^2^
*VEGFA*	4.6	0.52	<0.001 ^2^	4.52	1.36	0.004 ^2^
*CCL2*	9.39	0.87	<0.001 ^2^	4.93	2.91	0.243 ^1^
*PTGS2*	2.93	0.56	<0.001 ^1^	3.55	2.22	0.240 ^1^
*ACTA2*	4.57	1.91	0.008 ^1^	3.23	1.06	0.008 ^1^
*TJP1*	4.44	1.72	<0.001 ^1^	2.74	1.29	0.019 ^2^
*CLDN2*	3.36	0.19	<0.001 ^1^	13.4	0.3	<0.001 ^1^
*IL7*	1.93	0.89	0.095	1.9	1.17	0.345 ^1^
*IL7Ra*	1.86	0.53	0.047 ^2^	3.06	1.04	0.037 ^1^
*IL10*	0.78	0.70	0.805 ^2^	2.16	2.73	0.695 ^2^
*IL10Ra*	0.56	0.56	0.993 ^2^	1.57	0.97	0.245 ^2^

Data presented as geometric means of normalized relative quantities (NRQ) and analyzed as log-transformed data using ^1^*t*-test for independent samples or ^2^*t*-test for independent samples with Welch correction.

**Table 4:** Independent predictors of gene expression in non-cancerous tissues from GC patients—results of least squares multiple regression.

Explained Variable	Explanatory Variables		
	Entered	Retained	*R* ^2^
*CLDN2*	*BCLxL*, *SLC2A1*, *Ki67*, *NOS2*	*Ki67*: *r*_p_ = 0.81, *NOS2*: *r*_p_ = 0.80	0.827
*ACTA2*	*CCL2, PTGS2, IL-7, CDKN1A, TJP1*	*PTGS2*: *r*_p_ = 0.62	0.381
*TJP1*	*ACTA2, HIF1A, VEGFA, BCLxL, SLC2A1, ODC1, CDKN1A, BCL2, Ki67*	*ACTA2*: *r*_p_ = 0.68, *HIF1A*: *r*_p_ = 0.67, *VEGFA*: *r*_p_ = 0.71	0.896
*ODC1*	*SLC2A1*, *HIF1A*, *BCLxL*, *CDKN1A*, *BCL2*, *Ki67*, *VEGFA*, *TJP1*	*SLC2A1*: *r*_p_ = 0.64, *HIF1A*: *r*_p_ = 0.88	0.938
*SLC2A1*	*BCLxL, HIF1A, CDKN1A, ODC1, BCL2, CLDN2, Ki67, VEGFA, TJP1*	*ODC1: r*_p_ = 0.85	0.715
*VEGFA*	*BCLxL, BCL2, CCL2, HIF1A, CDKN1A, ODC1, Ki67, TJP1*	*TJP1: r*_p_ = 0.84	0.712
*HIF1A*	*SLC2A1, BCL2, Ki67, VEGFA, TJP1, BCLxL, CCL2, ODC1, CDKN1A*	*BCLxL*: *r*_p_ = 0.80, *CCL2*: *r*_p_ = 0.90, *ODC1*: *r*_p_ = 0.95, *CDKN1A*: *r*_p_ = −0.81	0.987
*PTGS2*	*ACTA2, CDKN1A, BCL2, CCL2*	*CCL2*: *r*_p_ = 0.77	0.585
*CCL2*	*ACTA2*, *HIF1A*, *CDKN1A*, *Ki67*, *PTGS2*, *BCL2*, *IL7*	*PTGS2*: *r*_p_ = 0.53, *BCL2*: *r*_p_ = 0.69, *IL7*: *r*_p_ = −0.78	0.876
*Ki67*	*BCLxL, CLDN2, SLC2A1, HIF1A, ODC1, BCL2, VEGFA, TJP1*	*BCLxL: r*_p_ = 0.70 *CLDN2*: *r*_p_ = 0.58	0.753
*‘BCLxL*	*SLC2A1, HIF1A, ODC1, CDKN1A, BCL2, CLDN2, Ki67, VEGFA, TJP1*	*HIF1A*: *r*_p_ = 0.90	0.808
*BCL2*	*HIF1A, PTGS2, BCLxL, CCL2, SLC2A1, ODC1, CDKN1A, CLDN2, VEGFA, TPJ1, Ki67*	*HIF1A*: *r*_p_ = 0.75, *PTGS2*: *r*_p_ = 0.60	0.772
*CDKN1A*	*ACTA2, BCLxL, SLC2A1, BCL2, TJP1, PTGS2, CCL2, HIF1A, ODC1, VEGFA*	*CCL2*: *r*_p_ = 0.89, *HIF1A*: *r*_p_ = −0.73, *ODC1*: *r*_p_ = 0.59, *VEGFA*: *r*_p_ = 0.66	0.871

Variables significantly correlated with explained variable in univariate analysis (Pearson correlation) were entered into the least squares multiple regression analysis (listed as Explanatory Variables: Entered). Variables were retained (listed as Explanatory Variables: Retained) in the regression model if *p* < 0.1. Partial correlation coefficients (*r*_p_) are presented for explanatory variables independently from other variables associated with explained variable. Model fit is presented as the coefficient of determination (*R*^2^).

**Table 5:** Independent predictors of gene expression in tumor tissues from GC patients—results of least squares multiple regression.

Explained Variable	Explanatory Variables		
	Entered	Retained	*R* ^2^
*CLDN2*	*Ki67*, *BCLxL*	*BCLxL*: *r*_p_ = 0.80	0.642
*ACTA2*	*BCL2, CCL2, NOS2, CDKN1A, TJP1*	*BCL2*: *r*_p_ = 0.63	0.391
*TJP1*	*CCL2, IL7, NOS2, ODC1, ACTA2, BCL2, HIF1A, CDKN1A, PTGS2*	*BCL2*: *r*_p_ = 0.84, *HIF1A*: *r*_p_ = 0.71, *CDKN1A*: *r*_p_ = 0.91, *PTGS2*: *r*_p_ = −0.82	0.978
*ODC1*	*HIF1A*, *BCLxL*, *CCL2*, *SLC2A1*, *Ki67*, *CDKN1A*, *PTGS2*, *VEGFA*, *TJP1*	*HIF1A*: *r*_p_ = 0.86	0.736
*SLC2A1*	*Ki67, BCLxL, HIF1A, VEGFA, ODC1*	*Ki67*: *r*_p_ = 0.80	0.632
*VEGFA*	*BCLxL, ODC1, SLC2A1, HIF1A, Ki67, CDKN1A*	*SLC2A1*: *r*_p_ = 0.77	0.589
*HIF1A*	*CCL2, ODC1, BCL2, BCLxL, Ki67, PTGS2, VEGFA, TJP1, SLC2A1, IL7, CDKN1A*	*CCL2*: *r*_p_ = 0.78, *ODC1*: *r*_p_ = 0.76	0.896
*PTGS2*	*BCLxL, CCL2, VEGFA, HIF1A, Ki67, CDKN1A, ODC1, TJP1*	*BCLxL*: *r*_p_ = −0.60, *CCL2*: *r*_p_ = 0.85, *VEGFA*: *r*_p_ = 0.87	0.907
*CCL2*	*HIF1A, BCL2, IL7, ODC1, PTGS2, CDKN1A, ACTA2, TJP1*	*HIF1A*: *r*_p_ = 0.74, *BCL2*: *r*_p_ = 0.56	0.829
*Ki67*	*BCLxL, ODC1, CLDN2, HIF1A, PTGS2, CDKN1A, VEGFA, SLC2A1*	*BCLxL*: *r*_p_ = 0.91, *ODC1*: *r*_p_ = 0.82	0.940
*BCLxL*	*CLDN2, Ki67, SLC2A1, HIF1A, ODC1, VEGFA*	*CLDN2*: *r*_p_ = 0.72, *Ki67*: *r*_p_ = 0.87	0.913
*BCL2*	*TJP1, CCL2, CLDN2, HIF1A, IL7, CDKN1A, ACTA2*	*TJP1*: *r*_p_ = 0.88	0.779
*CDKN1A*	*PTGS2, TJP1, BCL2, CCL2, HIF1A, Ki67, NOS2, ODC1, VEGFA, ACTA2*	*PTGS2*: *r*_p_ = 0.76, *TJP1*: *r*_p_ = 0.89	0.915

Variables significantly correlated with explained variable in univariate analysis (Pearson correlation) were entered into the least squares multiple regression analysis (listed as Explanatory variables: Entered). Variables were retained (listed as Explanatory variables: Retained) in the regression model if *p* < 0.1. Partial correlation coefficients (*r*_p_) are presented for explanatory variables independently from other variables associated with explained variable. Model fit is presented as coefficient of determination (*R*^2^).

**Table 6:** Impact of pathological stage on fold change in expression of cancer-related genes in gastric cancer.

Gene	TNM ^1^	T ^1^	N (N0 vs. N1/2) ^2^	M ^2^	G ^1^
*Ki67*	0.63 ^3^	0.50 ^5^	0.77 vs. 3.16 ^3^	ns	ns
*BCL2*	ns	0.50 ^5^	0.11 vs. 0.62 ^5^	ns	ns
*BCLxL*	ns	ns	0.62 vs. 1.05 ^5^	ns	ns
*CDKN1A*	0.51 ^3^	0.55 ^3^	ns	0.50 vs. 1.44 ^5^	0.58 ^3^
*ODC1*	0.58 ^3^	0.46 ^5^	0.32 vs. 0.99 ^4^	ns	ns
*HIF1A*	0.56 ^3^	ns	0.43 vs. 1.39 ^4^	ns	ns
*CCL2*	ns	0.53 ^3^	0.22 vs. 0.8 ^5^	ns	0.54 ^3^
*PTGS2*	ns	ns	ns	ns	0.48 ^5^
*TJP1*	0.66 ^4^	0.69 ^4^	0.3 vs. 0.88 ^3^	0.49 vs. 1.59 ^5^	ns
*IL10*	ns	ns	ns	ns	0.56 ^3^

Data presented as ^1^ Spearman correlation coefficients (ρ) or ^2^ mean fold change in expression (tumor to adjacent) analyzed using one-way ANOVA on log-transformed data. Only significant associations or tendencies (*p* < 0.1) are presented. TNM, cancer stage (tumor-node-metastases); T, extension of primary tumor; N, lymph node involvement; M, distant metastases; G, histological grade; ns, non-significant. Statistical significance is marked as ^3^, *p* ≤ 0.05; ^4^, *p* < 0.01; ^5^, tendency (0.1 > *p* > 0.05).

**Table 7:** Characteristics of study population for analysis of systemic cytokine signatures.

Characteristics:	Benign	EC	GC		*p*
			Cardia	Non-Cardia	
*n*	39	92	32	32	-
Sex (F/M), n	18/21	31/61	7/25	10/21	0.195 ^1^
Age (yrs.), mean ± SD	61.1 ± 13	62.4 ± 9	62.3 ± 9	63.3 ± 11	0.820 ^2^
Stage (I/II/III/IV)		6/23/25/38	0/5/6/21	1/9/6/16	0.260 ^1^
Primary tumor, T (1/2/3/4)		8/15/27/42	0/1/8/23	1/2/12/17	0.059 ^1^
Lymph node metastasis, N (no/yes)		33/59	5/27	10/22	0.102 ^1^
Distant metastasis, M (no/yes)		54/38	11/21	16/16	0.058 ^1^

*n*, number of observations; F/M, female-to-male ratio; yrs., years; SD, standard deviation; ^1^, Chi-squared test; ^2^, one-way ANOVA; EC, esophageal squamous cell carcinoma; GC, gastric adenocarcinoma.

**Table 8:** Systemic concentration of cytokines, chemokines and growth factors in gastric cancer as compared to esophageal cancer and benign conditions of upper gastrointestinal tract.

Cytokine (pg/mL)	Benign Conditions	Esophageal Cancer	Gastric Cancer	*p* Value
IL-1β	0.15 (0.15–0.15) ^2,3^	0.15 (0.15–1.66) ^1,3^	1.12 (0.15–1.55) ^1,2^	<0.0001
IL-1ra	339.3 (116–630) ^2,3^	119.4 (71–518) ^1,3^	67.8 (42–167) ^1,2^	<0.0001
IL-4	3.52 (2.4–4.2) ^3^	2.97 (2.0–4.0) ^3^	4.50 (3.3–5.0) ^1,2^	<0.0001
IL-5	5.43 (4.1–7.5)	5.65 (3.8–7.0)	4.68 (3.0–8.1)	0.270
IL-6	5.31 (1–11) ^2,3^	8.84 (15.5–14) ^1^	8.12 (5.4–15) ^1^	0.010
IL-7	8.02 (5.5–10)	8.05 (6–11)	7.06 (4.8–9.5)	0.091
IL-8	43.5 (20–50)	33.6 (17–49)	21.7 (13–49)	0.058
IL-9	58.5 (12–70)	14.9 (7.5–69) ^3^	52.6 (38–67) ^2^	0.054
IL-10	4.15 (2.3–8.1)	4.83 (3.1–7.9)	4.33 (2.6–8.2)	0.817
IL-12(p70)	54.7 (28–81) ^3^	37.6 (18–70) ^3^	17.9 (6.9–57) ^1,2^	<0.001
IL-13	10.35 (5.6–14) ^3^	11.04 (7.6–16) ^3^	6.4 (4.3–9.8) ^1,2^	<0.0001
IFNγ	25.2 (16–34) ^2,3^	31.8 (19–47) ^1,3^	56.5 (28–74) ^1,2^	<0.0001
IP-10	925 (625–1244)	745 (490–1124)	831 (649–1097)	0.326
EOX1	135.5 (110–184)	138.8 (92–195)	150.5 (104–181)	0.888
FGF2	23.9 (13–30)	16.6 (8.6–28) ^3^	28.3 (18–36) ^2^	0.002
G-CSF	33.9 (31–46) ^2,3^	43.6 (34–65) ^1^	41.0 (34–52) ^1^	0.024
GM-CSF	3.05 (0.68–7.28) ^2^	6.39 (1.65–12.6) ^1,3^	2.61 (0.06–8.89) ^2^	0.008
MCP-1	66.1 (42–76) ^3^	54.3 (27–71) ^3^	24.1 (15–61) ^1,2^	<0.0001
MIP-1α	1.49 (0.2–2.7) ^2^	2.38 (1.4–3.4) ^1^	1.84 (1.5–2.7)	0.019
MIP-1β	74.5 (54–96)	53.5 (32–85)	74 (42–103)	0.058
PDGF-BB	1436 (1171–1970) ^3^	1584 (1021–2406) ^3^	1909 (1245–2757) ^1,2^	0.032
RANTES	19,865 (912–21,922) ^2,3^	1893 (640–21,105) ^1^	2992 (1898–5199) ^1^	0.025
TNFα	30.1 (25–34)	27.0 (22–32)	30.6 (23–39)	0.137
VEGF-A	43.4 (28–103)	50.2 (26–88)	48.8 (23–118)	0.955

Data presented as medians with interquartile range and analyzed using Kruskal–Wallis *H* test with Conover post hoc test. ^1^, significantly different from benign conditions of upper gastrointestinal tract; ^2^, significantly different from esophageal cancer; ^3^, significantly different from gastric cancer.

**Table 9:** Individual cytokines and cytokine panels as differential biomarkers in gastric cancer.

Cytokine	AUC (95%CI), *p*	Sens. and Spec.	*J* Index	Cut-Off
IL-1β	0.75 (0.65–0.83), *p* < 0.001	70.3% and 84.6%	0.549	>0.15 pg/mL
IL-1ra	0.76 (0.66–0.84), *p* < 0.001	81.2% and 61.5%	0.428	≤238.1 pg/mL
IL-4	0.68 (0.58–0.76), *p* = 0.001	57.8% and 76.9%	0.347	>4.15 pg/mL
IL-6	0.67 (0.57–0.76), *p* = 0.003	75.0% and 53.8%	0.289	>5.53 pg/mL
IL12(p70)	0.71 (0.61–0.79), *p* < 0.001	62.5% and 74.4%	0.369	≤29.3 pg/mL
IL-13	0.66 (0.56–0.75), *p* = 0.003	79.7% and 51.3%	0.310	≤10.3 pg/mL
IFNγ	0.76 (0.66–0.84), *p* < 0.001	54.7% and 94.9%	0.496	>54.01 pg/mL
G-CSF	0.62 (0.52–0.71), *p* = 0.043	82.8% and 41.0%	0.238	>32.3 pg/mL
MCP-1	0.72 (0.62–0.80), *p* < 0.001	62.5% and 76.9%	0.394	≤44.4 pg/mL
PDGF-BB	0.64 (0.54–0.73), *p* = 0.012	53.1% and 74.4%	0.275	>1891 pg/mL
RANTES	0.67 (0.57–0.76), *p* = 0.007	78.1% and 66.7%	0.448	≤6005 pg/mL
Panel 1 ^1^	0.87 (0.79–0.93), *p* < 0.001	71.9% and 94.9%	0.668	>0.742 ^3^
Panel 2 ^2^	0.89 (0.82–0.95), *p* < 0.001	82.8% and 92.3%	0.751	>0.595 ^3^

^1^, Cytokine panel selected in logistic regression (stepwise method) consisting of IL-1β, IL-1ra, IL-6, and RANTES; ^2^, cytokine panel selected in logistic regression (backward method) consisting of IL-1ra, IL-4, IL-13, and IL-6; ^3^, predicted probabilities. AUC, area under receiver operating characteristics (ROC) curve; CI, confidence interval; sens., sensitivity; spec., specificity; J index, Youden index.

**Table 10:** Primers’ sequences.

Symbol	Gene Name	Accession No.	Primer Sequence 5′→3′	Amp. Size (bp)
*IL7* ^1^	Interleukin 7	NM_000880.4	F: gacagcatgaaagaaattggtagc R: caacttgcgagcagcacggaat	117
*IL7Ra* ^1^	Interleukin 7 receptor alpha	NM_002185.5	F: atcgcagcactcactgacctgt R: tcaggcactttacctccacgag	101
*IL10* ^1^	Interleukin 10	NM_000572.3	F: tctccgagatgccttcagcaga R: tcagacaaggcttggcaaccca	126
*IL10Ra* ^1^	Interleukin 10 receptor alpha	NM_001558.4	F: gccgaaagaagctacccagtgt R: ggtccaagttcttcagctctgg	153
*ACTA2* ^1^	Alpha smooth muscle actin	NM_001141945.2	F: ctatgcctctggacgcacaact R: cagatccagacgcatgatggca	115
*BCL2* ^1^	B-cell lymphoma 2	NM_000633.3	F: atcgccctgtggatgactgagt R: gccaggagaaatcaaacagaggc	127
*BCLxL* ^1^	B-cell lymphoma-extra large	NM_001317919.2	F: gccacttacctgaatgaccacc R: aaccagcggttgaagcgttcct	131
*CCL2* ^1^	Monocyte chemoattractant protein 1 (MCP1)	NM_002982.4	F: agaatcaccagcagcaagtgtcc R: tcctgaacccacttctgcttgg	98
*CDKN1A* ^1^	Cyclin Dependent Kinase Inhibitor 1A (p21^CIP1/WAF1^)	NM_001220777.2	F: aggtggacctggagactctcag R: tcctcttggagaagatcagccg	95
*GAPDH* ^2^	Glyceraldehyde-3-phosphate dehydrogenase	NM_001256799.3	F: tagattattctctgatttggtcgtattgg R: gctcctggaagatggtgatgg	223
*CLDN2* ^1^	Claudin 2	NM_020384.4	F: gtgacagcagttggcttctcca R: ggagattgcactggatgtcacc	153
*SLC2A1* ^1^	Glucose transporter 1 (GLUT1)	NM_006516.4	F: ttgcaggcttctccaactggac R: cagaaccaggagcacagtgaag	113
*HIF1A* ^1^	Hypoxia-inducible factor 1α	NM_181054.3	F: tatgagccagaagaacttttaggc R: cacctcttttggcaagcatcctg	145
*Ki67* ^1^	Proliferation marker Ki67	NM_001145966.2	F: gaaagagtggcaacctgccttc R: gcaccaagttttactacatctgcc	151
*NOS2* ^1^	Inducible nitric oxide synthase	NM_000625.4	F: gctctacacctccaatgtgacc R: ctgccgagatttgagcctcatg	136
*ODC1* ^1^	Ornithine decarboxylase	NM_001287189.2	F: ccaaagcagtctgtcgtctcag R: cagagattgcctgcacgaaggt	162
*PTGS2* ^1^	Prostaglandin-endoperoxide synthase 2 (COX2)	NM_000963.4	F: cggtgaaactctggctagacag R: gcaaaccgtagatgctcaggga	156
*TJP1* ^1^	Tight junction protein 1	NM_001355014.2	F: gtccagaatctcggaaaagtgcc R: ctttcagcgcaccataccaacc	132
*VEGFA* ^1^	Vascular endothelial growth factor A	NM_001025366.3	F: ttgccttgctgctctacctcca R: gatggcagtagctgcgctgata	126

Amp., amplicon; ^1^, primer sequences were as proposed by Origene (www.origene.com); ^2^, primers were designed using Beacon Designer Probe/Primer Design Software (BioRad), validated in silico by Blast analysis, and their specificity tested by means of melting curve analysis and an electrophoresis in a high-resolution agarose. Forward and reverse primer sequences are denoted by “F” and “R”, respectively.

## Abbreviations

AUC	Area under ROC curve
EC	Esophageal cancer
EMT	Epithelial-mesenchymal transition
GC	Gastric cancer
GERD	Gastro-esophageal reflux disease
IL	Interleukin
NRQ	Normalized relative quantity
ROC	Receiver operating characteristics curve analysis
TNM	Tumor-node-metastasis cancer staging system
